# Offering an American Graduate Medical HIV Course to Health Care Workers in Resource-Limited Settings via the Internet

**DOI:** 10.1371/journal.pone.0052663

**Published:** 2012-12-20

**Authors:** Michael H. Chung, Anneleen O. Severynen, Matthew P. Hals, Robert D. Harrington, David H. Spach, H. Nina Kim

**Affiliations:** 1 Department of Global Health, University of Washington, Seattle, Washington, United States of America; 2 Department of Medicine, University of Washington, Seattle, Washington, United States of America; 3 Department of Epidemiology, University of Washington, Seattle, Washington, United States of America; Cincinnati Childrens Hospital Medical Center, United States of America

## Abstract

**Background:**

Western accredited medical universities can offer graduate-level academic courses to health care workers (HCWs) in resource-limited settings through the internet. It is not known whether HCWs are interested in these online courses, whether they can perform as well as matriculated students, or whether such courses are educationally or practically relevant.

**Methods and Findings:**

In 2011, the University of Washington (UW) Schools of Medicine and Nursing offered the graduate course, “Clinical Management of HIV”, to HCWs that included a demographic survey, knowledge assessment, and course evaluation. UW faculty delivered HIV clinical topics through ten 2-hour weekly sessions from the perspectives of practicing HIV medicine in developed and developing settings. HCWs viewed lectures through Adobe Acrobat Connect Pro (Adobe Systems, San Jose, CA), and completed online homework on HIV Web Study (http://depts.washington.edu/hivaids/) and online quizzes. HCWs, who met the same passing requirements as UW students by attending 80% lectures, completing ≥90% homework, and achieving a cumulative ≥70% grade on quizzes, were awarded a certificate. 369 HCWs at 33 sites in 21 countries joined the course in 2011, a >15-fold increase since the course was first offered in 2007. The majority of HCWs came from Africa (72%), and most were physicians (41%), nurses (22%), or midlevel practitioners (20%). 298 HCWs (81%) passed all requirements and earned a certificate. In a paired analysis of pre- and post-course HIV knowledge assessments, 56% of HCWs improved their post-course score (p<0.0001) with 27% improving by at least 30%. In the course evaluation, most HCWs rated the course as excellent (53%) or very good (39%).

**Conclusions:**

This online HIV course demonstrated that opening a Western graduate medical and nursing curriculum to HCWs in resource-limited settings is feasible, popular, and valuable, and may address logistic and economic barriers to the provision of high quality education in these settings.

## Introduction

The availability of effective antiretroviral therapy and the rapid expansion of HIV treatment clinics in regions such as sub-Saharan Africa have increased the demand for HIV medical training [1], [2], [3]. In response, national HIV agencies and institutions have developed in-service standardized short courses to train large groups of health care workers (HCWs) [4]. The quality of the lecturers and presentations in these courses varies according to local capacity, and the method of teaching is primarily didactic and focused on application of national guidelines [3], [5].

Distance learning technology provides an opportunity for HCWs in resource-limited settings to access international HIV medical expertise without having to leave their countries [6]. There are, however, few opportunities for HCWs to participate in graduate-level clinical courses offered by accredited medical and nursing schools in the U.S. and Europe. These academic courses usually take several months to complete, maintain high standards for passing, and require that prospective students travel and temporarily reside in these countries. If such graduate courses were made freely available online, it is not known whether HCWs in resource-limited countries would enroll and could successfully complete the course requirements or whether these courses would be educationally or practically relevant.

Since 2007, the University of Washington (UW) Schools of Medicine and Nursing have opened the winter quarter HIV clinical course, “Clinical Management of HIV,” to participation from HCWs living in resource-limited countries. This graduate-level course teaches evidence-based HIV medicine through weekly web-cast lectures, online case-based homework, and multiple-choice quizzes, and maintains the same passing requirements for international HCWs as for UW students enrolled in Seattle. This paper describes the implementation of the course, its acceptance among HCWs, and its effectiveness teaching clinical HIV medicine.

## Methods

### Ethics Statement

Prior to the start of the study, it was determined that the study did not require Institutional Review Board (IRB) review or approval from the UW, because: 1) private information was not collected specifically for a research project through an interaction or intervention with living individuals; and 2) investigators could not readily ascertain the identity of the individuals to whom the coded private information pertained because direct and indirect identifiers were removed from the dataset and destroyed before research data analysis began. Given this information and the definition of “human subject” under 45 CFR 46.102(f), it was concluded that the study did not meet the federal regulatory definition of “human subjects research.” As a result, all of the data was analyzed anonymously and written informed consent was not obtained.

**Figure 1:**
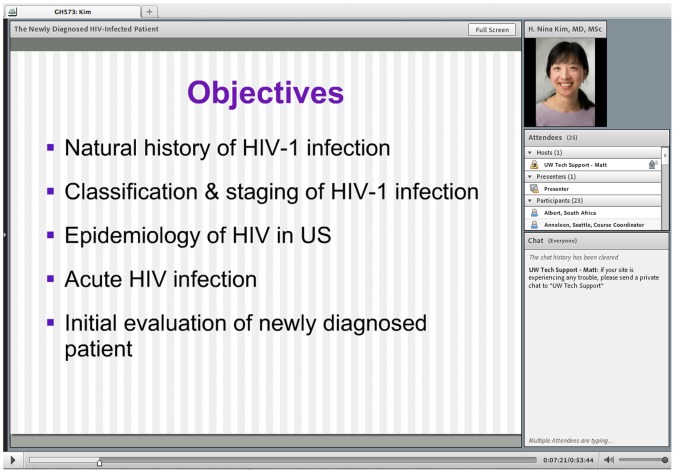
Adobe Connect Lecture Screenshot.

The subject in Figure 1 gave written informed consent, as outlined in the PLoS consent form, to publication of her photograph.

### Course

The UW graduate-level course, Clinical Management of HIV [Global Health (GH) 573], has been offered conjointly by the UW Schools of Medicine and Nursing to matriculated UW students and international HCWs every winter quarter of the academic calendar, between January and March, since 2007. The course is composed of ten two-hour presentations that are delivered once per week by UW School of Medicine faculty in Seattle, Washington, USA. There are two hour-long lecturers per session; the first lecturer addresses the topic from the perspective of practicing HIV medicine in the U.S. and the second from the perspective of practicing in a resource-limited country. Lecturers speaking from the perspective of practicing HIV medicine in resource-limited countries are composed primarily of U.S. faculty who have lived and treated HIV patients in these conditions, but also include regional experts from these settings.

Topics include the initial evaluations of an HIV patient, the diagnosis and treatment of opportunistic infections, and multiple aspects of antiretroviral therapy (Table 1). Lecturers use case-based teaching, present a variety of clinical scenarios, and discuss the literature and scientific evidence behind clinical decision-making and suggested guidelines. American HIV clinical practices are regularly compared and contrasted with the national guidelines of resource-limited countries.

**Table 1:** Weekly Lecture Topics and Objectives.

**Diagnosing HIV and the Initial Evaluation of HIV-infected Patient**
Conduct an initial history & physical of a newly diagnosed HIV-infected patient
Stage patients’ HIV disease severity based on both CDC and WHO HIV classifications
Describe the rationale and indications for HIV testing
**Introduction to Antiretroviral Therapy**
Describe the goals of antiretroviral therapy, particularly the motivation behind the public health approach
Identify the distinguishing features of each class of antiretrovirals, as well as the pros/cons of different regimens
List the indications for starting antiretroviral therapy, based on US as well as WHO guidelines
**Antiretrovirals: Adverse Effects, Drug Resistance, and Drug Interactions**
Describe the adverse effects of antiretroviral agents – both common and severe
Outline the indications for HIV resistance testing as well as the approach in resource-limited settings when such testing is not available
**Opportunistic Infections: Non-neurology, Non-tuberculosis**
Outline the diagnosis and management of Pneumocystis jiroveciipneumonia, disseminated Mycobacterium avium complex, Candidal esophagitis, and Cytomegalovirus disease in patients with AIDS
Explain the key and expanded role of co-trimoxazole prophylaxis in the resource-limited setting
Recognize and define immune reconstitution syndrome
**Opportunistic Infections: Tuberculosis, Cryptococcus, Toxoplasmosis and Progressive Multifocal Leukoencephalopathy**
Outline the diagnostic algorithm for focal and non-focal brain lesions and for meningitis in patients with advanced HIV
Describe the management of cerebral toxoplasmosis, progressive multifocal leukoencephalopathy, and Cryptococcal meningitis
Explain the interaction between HIV and TB, and the principles of TB treatment in the HIV-infected patient
**HIV-associated Malignancies & Dermatology**
Identify the key clinical features of the major AIDS-defining malignancies: Kaposi’s sarcoma, Non-Hodgkin’s lymphoma, Primary CNS lymphoma
Recognize and recall common themes in HIV-related skin disease
**Management of Sexually Transmitted Infections in HIV-infected Patients**
Identify the most common STI syndromes
Differentiate between the syndromic versus etiologic approach to management of STIs in HIV-infected patients
**HIV Vertical Transmission and Pregnancy**
Discuss the risk factors for mother-to-child HIV transmission (MTCT), as well as the interventions to prevent MTCT in both US and resource-limited settings
**Pediatric HIV**
Contrast the natural history and manifestations of HIV in infants and children from that of adults
Describe the unique challenges in diagnosis and management in pediatric HIV, particularly from global perspective
**Post-exposure Prophylaxis for HIV and Preventive Care**
Outline the strategies to reduce secondary HIV transmission, starting with the infected patient
Recognize the indications for post-exposure prophylaxis for both occupational and sexual HIV exposures

The live lectures in Seattle are broadcast and recorded using Adobe Acrobat Connect Pro v8.2 (Adobe Systems, San Jose, California, USA), an internet-based web platform that transmits video of the PowerPoint slides and audio of the lecturer’s voice. Video capture of the lecturer and the classroom audience is disabled to reduce file size and decrease bandwidth requirements. Students participating synchronously from distant sites interact directly with lecturers by typing questions and comments into a chat box (Figure 1). Lectures are recorded and archived online for all students.

**Figure 2:**
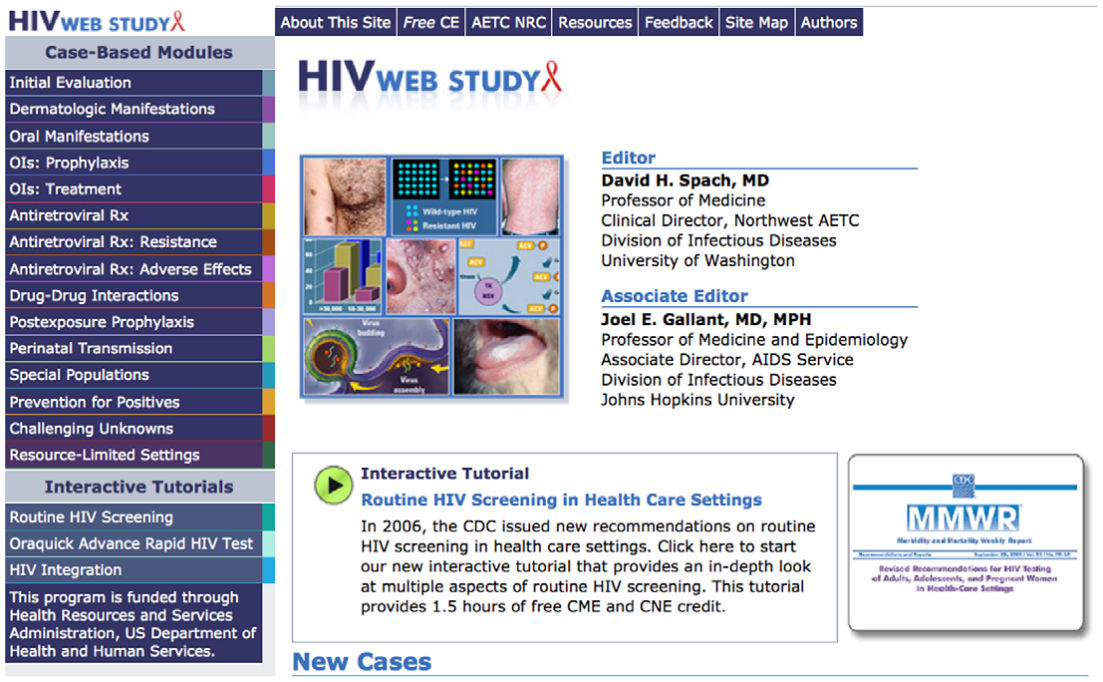
HIV Web Study.

Online homework is composed of case-based modules from HIV Web Study (http://depts.washington.edu/hivaids/) (Figure 2), a free online HIV educational service provided by the Northwest AIDS Education and Training Center (AETC) and the UW. Targeting HIV practitioners in the Pacific Northwest and throughout the U.S., HIV Web Study contains more than 60 American-based HIV clinical case studies, with multiple-choice questions and an in-depth discussion of the topic referencing peer-reviewed literature. Based on their relevance to the HIV topic covered in the lecture, four to five HIV Web Study cases are chosen and associated with each week’s lecture and assigned as homework.

Students register online through Catalyst Web Tools, a UW learning management software that forms an online class workspace (www.washington.edu/lst/web_tools). Each student receives an individual username and password and logs in through the Catalyst website to access the course syllabus, the week’s lectures, PowerPoint slides, associated readings, homework assignments, and quizzes. Online weekly quizzes assess learning through 6 to 7 multiple-choice questions that are generally drawn evenly from both the U.S.-based and resource-limited-based lectures and the results of which are automatically graded and recorded by Catalyst. The site also tracks each student’s progress in completing the online homework.

The HIV online course is offered free of cost to HCWs in resource-limited countries. HCWs at these sites register online as international students and are organized into classes that participate either synchronously (at the same time as the lecture given in Seattle) or asynchronously (at an alternative time and day more convenient to local students due to time zone differences). Prospective students learn of the course through word of mouth, web searches, and from introductory emails sent to students, sites, and organizations that have previously participated in the course or have been associated with the University of Washington. Classes are led by a volunteer site coordinator and organized into groups of no fewer than 5 students in order to stimulate learning through discussion and interaction at asynchronous sites. The site coordinator organizes the classroom, downloads the lecture, records attendance, and ensures that students are able to access the course website to complete the homework and take the quizzes. Distant site set-up requires: classroom space, internet access, a computer with external speakers, and, if class sizes are large, a projector. Asynchronous sites download a recorded version of the lecture and are encouraged to identify a clinical facilitator who can promote local discussion and answer questions during the lecture. Clinical facilitators are identified by local sites and selected based on their HIV clinical experience, medical training, seniority, and ability to support the course.

The course is aimed at students with basic proficiency in taking a clinical history and performing a physical examination. At the UW, this basic proficiency and an undergraduate degree are necessary pre-requisites to enroll in this otherwise introductory-level graduate course. Eligible participants include physicians, nurses, clinical officers and nurse practitioners (midlevel practitioners), pharmacists, and students/resident/fellows of medicine, nursing, public health, and pharmacy. Students who attend ≥80% of the lectures, complete ≥90% of the homework, and have a cumulative quiz grade ≥70% earn a certificate from the UW. Twelve optional online homework assignments can boost cumulative quiz grades by 1% for each assignment that is successfully completed. The same eligibility and passing requirements for the course are applied to both UW and international students.

### 2011 International Student Survey, Knowledge Assessment, and Course Evaluation

In 2011, international students were asked to complete an online survey, participate in a pre- and post-course assessment of HIV knowledge, and provide a course evaluation. The survey was performed during online course registration and queried the student regarding their workplace setting, professional background, HIV clinical experience, and access to training opportunities. The pre- and post-course HIV knowledge assessment was delivered by email to all international students. The assessment consisted of ten multiple-choice case-based HIV questions, and was given before course initiation and then, after scrambling the order of the same questions, four months after course completion (Text S1). Each question provided four multiple-choice answers of which only one was correct. Knowledge assessments were linked to the student login name to allow pairwise t-test statistical comparisons. After the course, an anonymous online course evaluation asked both domestic and international students to rate aspects of the course on a scale from 0 (very poor) to 5 (excellent) and to compare it to other courses on a scale from 0 (much lower) to 6 (much higher).

## Results

### Course and Student Demographics

In January 2007, HCWs from Haiti and Kenya were the first international participants to join students in Seattle and participate in the GH 573 course. Enrollment of HCWs from international sites increased every year since then and in 2011 included students in 33 sites within 21 different countries (Table 2). Of the 33 sites participating online, 11 sites (enrolling 27% of the total number of international students) joined synchronously and 22 sites (enrolling 73% of students) joined asynchronously. In 2011, 369 HCWs enrolled in the course and of these 289 (76%) completed the international student survey at registration. The majority of international students came from Africa (202; 72%), followed by the Caribbean (54; 19%), Asia (14; 5%), and Latin America (9; 2%). Most of the participants (68%) disclosed that there were no other HIV training opportunities in their area.

**Table 2:** Sites and countries by year.

Year	Countries (sites)	Sites	Students
2007	Haiti, Kenya	2	24
2008	Ethiopia, Haiti, India, Jamaica, Kenya, Peru (4), Tanzania	10	74
2009	Botswana, Ethiopia (2), India, Jamaica, Kenya, Malawi, Namibia, Peru (3), Saint Lucia, Trinidad and Tobago	13	154
2010	Anguilla, British Virgin Islands, Dominica, Ethiopia (3), Grenada, India, Jamaica (2), Kenya,Montserrat, Mozambique, Namibia (3), Peru (2), Saint Kitts and Nevis (2), Saint Lucia,Saint Vincent and the Grenadines	22	249
2011	Antigua and Barbuda, Botswana, British Virgin Islands, Dominica, Ethiopia (2), Grenada, India,Jamaica, Kenya (6), Mozambique, Namibia (5), Peru (2), Saint Kitts and Nevis, Saint Lucia, Somalia,South Africa, Tanzania (2), Trinidad and Tobago, Tunisia, Vietnam, Zambia	33	369

Based on the 2011 survey, international participants included physicians (41%), nurses (22%), midlevel practitioners (20%), midwives (5%), pharmacists (5%), and other HCWs (7%). Most respondents (42%) worked primarily in a hospital environment, including outpatient clinics (25%) and inpatient wards (17%). The remaining worked at non-governmental organizations (25%), academic institutions (14%), community clinics (11%), or other types of facilities (8%). The majority (55%) cared for at least 20 HIV-infected patients per month, and 63% reported having been engaged in clinical practice for at least 5 years. Almost half (48%) described previous classroom training in HIV clinical management and, in terms of confidence in their knowledge of HIV care, 41% graded themselves as “mostly confident” and 12% as “very confident.”

Of the 369 international students who enrolled in the 2011 course, 298 (81%) met passing requirements and earned a certificate. The median number of lectures attended was 9 [interquartile range (IQR), 8–10]; 331 (89%) students attended 8 or more of the lectures and 310 (84%) completed ≥90% of the homework. The median cumulative quiz score was 78% (IQR, 62–88%). While 241 (65%) students scored a cumulative quiz score ≥70%, 57 (15%) completed optional homework assignments to raise their score to this passing level. There were no statistically significant differences in quiz scores and passing rates between international students by type of online participation (synchronous vs. asynchronous), professional background, or workplace setting.

In 2011, there were 14 UW matriculated graduate students who took the course in Seattle. Nine students were enrolled in the School of Nursing, 2 in Pharmacy, 2 in Medicine, and 1 in Public Health. All 14 students passed the course with a median cumulative quiz score of 82% (IQR, 76–90%).

### Knowledge Assessment

Of the 298 international students who passed the course, 126 (42%) students completed both the pre-course and post-course HIV knowledge assessments. There were no statistically significant differences in country of origin, professional background, workplace setting, or years of clinical experience between those international students who completed the pre- and post-course knowledge assessments and those who did not. In the pre-course assessment, 96 (76%) students scored less than 70% compared to 71 (56%) students in the post-course assessment. In a paired analysis, 71 (56%) students improved their score on the post-course assessment (p<0.0001), and of these 31 (25%) improved their score by at least 30%. Of the 55 (44%) students who did not improve, 34 (27%) scored lower in the post-course HIV knowledge assessment than in the pre-course assessment and 21 (17%) scored the same.

Among the 126 students who took both the pre-course and post-course HIV knowledge assessments, information on professional backgrounds was available for 86 (68%) students. Of these students, 34 (40%) were physicians, 25 (29%) were nurses, 20 (23%) were midlevel practitioners, and 7 (8%) were other HCWs. Thirteen (38%) physicians, 4 (16%) nurses and 6 (30%) midlevel practitioners scored ≥70% on the pre-course assessment, while 19 (56%) physicians, 7 (28%) nurses, and 10 (50%) midlevel practitioners performed ≥70% on the post-course assessment. Twenty-one (62%) physicians scored better on their post-course knowledge assessment compared to 8 (32%) nurses and 12 (60%) midlevel practitioners.

### Course Evaluation

Of the 383 domestic and international students who enrolled in the course, 207 (54%) completed the course evaluation. Most of the students rated the course as excellent (53%; score, 5) or very good (39%; score, 4) (overall course median score, 4.6; IQR, 4–5). In terms of the delivery format, 29% rated it as excellent and 46% as very good (median score, 4.0; IQR, 4–5). The lowest score of all measured variables was “availability of extra help when needed” (median score, 3.7; IQR, 3–4). The median number of hours spent by students per week on the course was 7.3. In comparison to other courses, using a scale from 0 (much lower) to 6 (much higher), students rated this course as more intellectually challenging (median score, 5.2; IQR, 4–6) and requiring greater effort (median score, 5.1; IQR, 5–6) and involvement (median score, 5.3; IQR 5–6).

## Discussion

This graduate-level academic course in HIV medicine offered by an American university successfully accommodated HCWs from resource-limited settings and significantly increased their HIV clinical knowledge through an online offering. Within 5 years, the course grew from 24 enrolled HCWs in 2 countries outside the U.S. to 369 HCWs at 33 sites in 21 countries. The same standards required of domestic matriculated students to attend class (≥80%), complete homework (≥90%), and pass quizzes (≥70%), were also expected of the international HCWs. Under these conditions, more than 80% of HCWs passed all course requirements, more than half (56%) improved their post-course knowledge assessment score, and nearly all (92%) rated the course as excellent or very good.

Distance learning technologies provide an opportunity for HCWs living in resource-limited countries to join students in classes offered at accredited American academic medical centers. As internet availability and broadband speeds increase around the world, the gap in access to quality educational resources is narrowing [7], [8]. Not only are lectures, speeches, books and journals available online, but entire courses, diplomas, and degree programs are now accessible [9]. Making available medical classrooms in the West leverages coursework that has already been developed, reviewed, and accredited, obviating the need to create entirely new courses for which there may be limited resources. Such online courses expand the reach of a quality academic medical education and can help meet the current demands of training the thousands of HCWs who are needed to treat the millions of people infected with HIV worldwide [10], [11].

American graduate medical training contrasts with the public health approach to care adopted by many developing countries in which clinicians are expected to learn and adhere to national guidelines and treatment algorithms [5]. Without a solid foundation of knowledge, like that provided in this course, HCWs may not understand the clinical evidence and reasoning behind evolving recommendations and guidelines that inevitably become outdated. Quality graduate medical and nursing courses contribute to training by teaching personalized clinical care, updating medical practices, and developing an individual’s capacity for evidence-based learning [12].

This course was unique in contrasting the different approaches to clinical management from the developed and developing world. Issues such as the inability to pay and the scarcity of diagnostic tools, medications, and treatment methodologies were regularly discussed, and led to a rich exchange of ideas and evolving attitudes on standards of clinical care. American students learned the value of physical examination skills in regions where expensive diagnostic equipment is unavailable. Similarly, exposure to American diagnostic and treatment methods expanded the knowledge of HCWs preparing for the future and seeking to understand what medical choices should be made at an individual and national strategic level. The approach of this course emphasized the need to understand the published clinical evidence behind varying national and regional guidelines and to realize that best clinical practices are made considering cost as well as medical outcomes.

Including online interactive homework and evaluative learning assessments significantly increased the pedagogical value of this online course. The assigned online homework was case-based and interactive; students had to read each case and choose answers before explanations were provided. The case discussions in HIV Web Study were thoroughly researched, detailed, and referenced, and the Catalyst website recorded students’ multiple choice answers and tracked homework completion. In addition, weekly evaluative quizzes compelled students to study and learn the material in order to receive a passing score. These characteristics substantially improved the educational impact of the course compared to one in which students simply listen to lectures alone.

Opening a graduate medical course to an online international audience creates an array of challenges that must be carefully considered including: ensuring uniform quality and support to disparate, resource-constrained sites worldwide, combining the needs of domestic and international students into a single virtual classroom, and understanding the infrastructure and financial support needed to replicate this course. First, since the course relied on volunteers at distant sites to establish the classroom, take attendance and download lectures, it was difficult to ensure uniform quality at all sites. Some sites were challenged by the inconsistency and speed of internet access, the availability of electricity, and the presence and functioning of computer equipment. Some volunteer coordinators were less computer and internet literate than others and required more technical assistance from Seattle. The ability of coordinators to facilitate clinical discussion, take accurate attendance, and prevent cheating could not be confirmed and could have influenced the educational quality and validity of student assessments.

Second, the educational backgrounds and HIV work experience of the international students differed from that of the domestic students who were mainly American graduate students in nursing, medicine, pharmacy, and public health, and were outnumbered by a ratio of 26 to 1. As HCWs, most of the international students had previous training in HIV and had been treating patients by the time they took the course. These differences influenced subject topics and levels of discussion. It is not clear that these changes benefited domestic students, although it appeared that exposure to the issues raised by international students enriched course content and discussions around the impact of cost on medical care.

Similarly, it should be noted that while 81% of international students passed the course, 15% did so only after completing the optional homework assignments, in contrast with the 82% of UW students who passed based on quiz scores alone. Thus, domestic and international students may have differed not only in terms of clinical experience but in educational skills and abilities. This is also reflected in the fact that the passing rate on knowledge assessments differed between doctors and nurses in international settings. Physicians and clinical officers performed better on the pre- and post-course knowledge assessments compared to nurses in this study. Thus, the benefits of a rigorous course such as this may not extend to health care workers of all backgrounds and educational experiences.

Finally, further study is necessary to identify the resources that are needed to make a graduate online course successful. A significant amount of the course’s education occurred through the online interactive homework performed on HIV Web Study. The HIV Web Study site was developed in 2004 and is supported with funding from the Health Resources and Services Administration, U.S. Department of Health and Human Services; the site is a free educational resource intended for HIV practitioners in the U.S. There was no added funding required to access and utilize the HIV Web Study course for the GH 573 course. While the interactive online homework taught important evidence-based clinical knowledge, it could not simulate clinical scenarios and treatment care approaches from every country represented in the course. In this way, the course cannot substitute for local HIV clinical training according to national guidelines, but rather is more helpful in deepening already existing knowledge and clinical skills.

The course infrastructure that was created for UW matriculated students was able to accommodate additional international students because much of the course was online and could be automated. However, international students did note in their evaluations that the “availability of extra help when needed” was the most challenging aspect of the course, suggesting that additional resources and mentors will be required for the course to expand to retain its integrity. The resources to develop interactive, online homework and manage a course that may attract hundreds or thousands of international students may not be easily replicated when offering graduate medical or nursing courses online.

A weakness of this analysis is that only a minority of international students (42%) completed both the pre- and post-course knowledge assessment to allow pairwise comparisons, and therefore our results may not be generalizable to all the students who took the course. Performance on this knowledge assessment also relied on a variety of factors that was not related to the course content including English language proficiency, multiple-choice test-taking skills, and a good internet connection. Finally, as the course evaluations were performed anonymously, it was not possible to compare assessments between UW and international students.

In conclusion, our online HIV course demonstrates that expanding enrollment in an accredited graduate school in America to include HCWs in resource-limited settings is feasible, popular, and valuable. The course provides a model for distance-based health curriculums with its access to lectures from expert faculty combined with an online interactive forum for self-study and assessment. Approaching HIV clinical care from peer-reviewed Western and resource-limited perspectives developed a capacity for evidence-based learning among HCWs and exposed them to diagnostic and treatment modalities that should eventually help raise the standard of care wherever they practice. Setting high standards to pass and earn a course certificate did not dissuade HCWs in resource-limited settings from enrolling in the course, which experienced a greater than 15-fold increase in enrollment over 5 years. To the contrary, the rigor and high standards of the course are what attracted many of the participants. With continued rapid growth in access to the internet, there is great potential for the already developed graduate medical and nursing curriculums in the West to quickly and directly meet the training needs of HCWs in resource-limited settings. Whether the financial and infrastructure support for these endeavors will be available is unknown but, as this study demonstrates, the demand and benefits are clear.

## Supporting Information

Text S1 **Knowledge Assessment.**
(DOCX)
