# Supplementary material for: Offering an American Graduate Medical HIV Course to Health Care Workers in Resource-Limited Settings via the Internet
Source: PLoS One. 2012 Dec 20;7(12):e52663. doi: 10.1371/journal.pone.0052663 (PMC3527561; doi:10.1371/journal.pone.0052663)
Supplement: Text S1 — Knowledge Assessment. (DOCX) [file pone.0052663.s001.docx]

Which of the following is TRUE regarding the natural history of HIV-1 and the opportunistic conditions that can occur?

1. HIV-1 viral level is not correlated with the progression to AIDS.
2. Tuberculosis can occur at any CD4 cell count level – though the risk of extrapulmonary infection increases with lower CD4 cell counts.
3. Only a minority of patients with acute HIV-1 infection have fever or lethargy.
4. Multiple studies suggest that HIV-infected patients who start antiretroviral therapy at higher CD4 cell counts experience reduced risk for non-AIDS-defining illnesses.
5. B and D.

A 43 year-old man presents with a 1-month history of progressive non-productive cough and dyspnea with exertion. He was found to be HIV-infected 5 years ago but has not been in care for the last 3 years because he felt well until now. Temperature is 38.2, respiratory rate 24, oxygen saturation is 93% on room air and drops to 88% with walking – PaO2 is 60 mmHg. Lab results show a mild pancytopenia and CD4 cell count of 43 cells/mm^3^. Chest radiograph reveals bilateral interstitial infiltrates. Which of the following is the best approach for this patient if resources are not an issue?

1. Start empiric therapy with high-dose oral trimethoprim-sulfamethoxazole therapy and concurrent corticosteroids. Continue this if patient shows improvement on return to clinic the next day.
2. Obtain induced sputum for *Pneumocystis jirovecii* as well as acid fast bacilli if indicated. Start high-dose intravenous trimethoprim-sulfamethoxazole therapy and concurrent corticosteroids before results return.
3. Given pancytopenia, avoid trimethoprim-sulfamethoxazole and choose clindamycin and primaquine instead.
4. Hold off on any empiric therapy until bronchoscopy can be arranged.

A 35 year-old woman with recently diagnosed HIV-1 infection presents to a clinic in Zambia with progressive right leg swelling and new plaques on her foot (shown in Figure). Which of the following is the most appropriate statement regarding her condition?


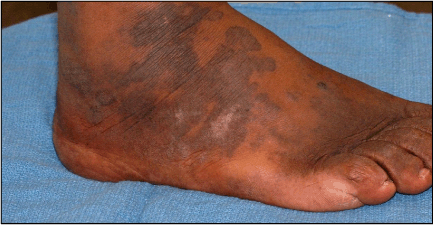


http://www.clinicaloptions.com

1. Bacillary angiomatosis is the more likely diagnosis since Kaposi’s sarcoma rarely occurs in women.
2. This condition will likely respond to a prolonged course of antifungal therapy.
3. Indications for treatment other than antiretroviral therapy for this condition include: symptomatic foot involvement, lymphedema and pulmonary or gastrointestinal disease.
4. Treatment of this condition in conjunction with highly-active antiretroviral therapy can be considered curative in all cases.

A 28 year-old HIV-infected woman presents for antenatal care in Kenya. She is about 10 weeks gestation and is not currently on antiretroviral therapy – her CD4 cell count last month was 550 cells/mm^3^. Which of the following statements is TRUE with regard to mother-to-child transmission of HIV infection in this patient?

1. The mother’s CD4 cell count is the most powerful predictor of HIV vertical transmission; so given her high CD4 cell count, antiretroviral therapy can be deferred.
2. Intrauterine transmission in the first trimester (rather than intrapartum or postpartum transmission) is responsible for the majority of cases of mother-to-child transmission of HIV so it is not clear that starting antiretroviral therapy now will be helpful.
3. If the patient is motivated to start therapy, the regimen recommended by the WHO for patients like her is zidovudine (AZT), lamivudine (3TC) and nevirapine (NVP).
4. On average in breastfeeding mothers, the risk of perinatal HIV transmission is reduced to <5% with highly-active antiretroviral therapy from a background risk of 35% or greater.

A 32 year-old HIV-infected man presents with new headache, nausea and vomiting. His CD4 cell count is 15 cells/mm^3^. He has been on Combivir and efavirenz for the past two months. Serum Cryptococcal antigen is positive with titer 1:1024. Non-contrast head CT is normal. Which of the following statements is correct?

1. Lumbar puncture can be deferred and antifungal therapy started given his positive serum Cryptococcal antigen.
2. Lumbar puncture should be performed and opening pressure measured to evaluate for increased intracranial pressure.
3. Dexamethasone, along with serial lumbar punctures, has been shown to benefit those patients with marked inflammation on cerebrospinal fluid.
4. Preferred first-line therapy for cryptococcal meningitis is fluconazole 800 mg PO once daily with flucytosine 100 mg/kg daily.

23 year-old man from Peru with HIV-1, CD4 cell count of 400 cells/mm^3^ on antiretroviral therapy with tenofovir, lamivudine and lopinavir-ritonavir (started 2 months ago) presents with new-onset cough, fevers and weight loss of 10 kg. His chest radiograph demonstrates a right lower lobe infilitrate and sputum returns positive for acid-fast bacilli. In addition to starting empiric therapy with isoniazid, rifampin, pyrazinamide and ethambutol, which of the following would be the most appropriate course of action?

1. Continue current antiretroviral therapy.
2. Discontinue antiretroviral therapy to reduce risk of immune reconstitution inflammatory syndrome.
3. Switch out the lopinavir-ritonavir for efavirenz.
4. Switch out the tenofovir for zidovudine to reduce risk of nephrotoxicity.

A 30 year-old woman presents to establish HIV care in a Ugandan clinic. She has never taken antiretroviral therapy and has no history of opportunistic infections. Her CD4 cell count is 335 cells/mm^3^. Choose the most appropriate course of action:

1. Initiation of antiretroviral therapy can be deferred since her CD4 cell count is still >300 cells/mm^3^.
2. She should be started on cotrimoxazole and discussion of antiretroviral therapy should begin given her CD4 cell count of <350 cells/mm^3^.
3. She should be started on cotrimoxazole, as well as stavudine, zidovudine and nevirapine at this visit.
4. She should be tested for chronic hepatitis B – if she is positive, then antiretroviral therapy is indicated.

A 43 year-old man with HIV infection presents to clinic with oral candidiasis. He has been on an initial antiretroviral regimen of zidovudine, lamivudine and efavirenz for the past 18 months. He admits that he has not been consistent about taking this regimen because of nausea and has missed on average 2-3 doses per week. You review his lab results and note that his CD4 cell count has remained ~70 cells/mm^3^ over the past year and he has been losing weight. HIV-1 viral load testing is still not available in your clinic and the set of antiretroviral agents available to you are: zidovudine, lamivudine, stavudine, didanosine, tenofovir, nevirapine, efavirenz, ritonavir, indinavir, nelfinavir, and lopinavir/ritonavir. In addition to reinforcing medication adherence with the patient, which of the following is the most appropriate course of action?

1. Continue current regimen and provide anti-nausea medication to improve adherence.
2. Switch out zidovudine for tenofovir since zidovudine was likely contributing to his persistent nausea. Continue lamivudine and efavirenz.
3. Switch out efavirenz for lopinavir/ritonavir since the patient is most likely to have developed resistance to efavirenz. Continue zidovudine and lamivudine.
4. Switch out efavirenz for lopinavir/ritonavir and add tenofovir to the regimen since the patient is likely to have developed resistance to lamivudine and efavirenz. Continue zidovudine and lamivudine.

A 42 year-old man presents for routine HIV care. He has been on the combination therapy of emtricitabine/tenofovir/efavirenz for the past 3 years with most recent CD4 cell count of 540 cells/mm^3^ and plasma HIV-1 RNA level below the limit of detection. His past history is unremarkable apart from an episode of gonorrhea during his 30s. He has a male partner who often accompanies him to the visits. Patient has no complaints. Which of the following statements is true regarding screening for sexually transmitted infections (STIs) in this patient?

1. Because he reports no genital symptoms, a syphilis serologic test should be sufficient screening.
2. You need additional information from the patient before determining a course of routine screening for STIs.
3. Because he is asymptomatic, no STI screening is necessary.
4. Because he has only one partner, no STI screening is necessary.

A 2 month-old infant is brought to a clinic in South Africa by his mother. She was recently diagnosed with HIV-1 infection. She says that the baby has been healthy and growing well. Which of the following statements is true regarding this infant?

1. The test of choice for diagnosing HIV-1 infection in this infant would be an HIV-1 ELISA.
2. The test of choice for diagnosing HIV-1 infection in this infant would be an HIV-1 p24 antigen.
3. If he is HIV-infected and has a CD4 cell percentage >25%, then antiretroviral therapy can be deferred.
4. If he is HIV-infected, then antiretroviral therapy is recommended regardless of clinical or immunologic status.
